# Role of the T2Dx magnetic resonance assay in patients with suspected bloodstream infection: a single-centre real-world experience

**DOI:** 10.1186/s12879-022-07096-w

**Published:** 2022-02-01

**Authors:** Angela Quirino, Vincenzo Scaglione, Nadia Marascio, Maria Mazzitelli, Eugenio Garofalo, Francesca Divenuto, Francesca Serapide, Andrea Bruni, Rosaria Lionello, Grazia Pavia, Chiara Costa, Aida Giancotti, Cinzia Peronace, Federico Longhini, Alessandro Russo, Maria Carla Liberto, Giovanni Matera, Carlo Torti, Enrico Maria Trecarichi

**Affiliations:** 1 Unit of Clinical Microbiology, Department of Health Sciences, “Magna Graecia” University of Catanzaro-“Mater Domini” Teaching Hospital, Catanzaro, Italy; 2 Unit of Infectious and Tropical Diseases, Department of Medical and Surgical Sciences, “Magna Graecia” University of Catanzaro-“Mater Domini” Teaching Hospital, Catanzaro, Italy; 3 Unit of Intensive Care, Department of Medical and Surgical Sciences, “Magna Graecia” University of Catanzaro-“Mater Domini” Teaching Hospital, Catanzaro, Italy; 4 “Magna Graecia” University of Catanzaro, Catanzaro, Italy; 5 “Mater Domini” Teaching Hospital, Catanzaro, Italy

**Keywords:** Blood stream infections, T2Dx, ESKAPE, Antimicrobial therapy

## Abstract

**Background:**

T2Dx was approved by the US Food and Drug Administration for the rapid detection of a modified panel of ESKAPE bacterial species or *Candida* spp. causing bloodstream infection (BSI).

**Patients and methods:**

We performed a retrospective, observational study from January 1, 2018 to December 31, 2019 of all hospitalised patients with suspected BSI who underwent assessment using T2Dx in addition to standard blood culture (BC). T2-positive patients (cases) were compared to a matched group of patients with BSI documented only by BC (1:2 ratio) to investigate the possible impact of T2Dx on the appropriateness of empirical antimicrobial therapy and 21-day mortality.

**Results:**

In total, 78 T2Dx-analysed samples (49 patients) were analysed. The T2Dx assay result was positive for18 patients and negative for 31 patients. The concordance rates of the T2Bacteria Panel and T2Candida Panel results with those of standard BC were 74.4% and 91.4%, respectively. In the matched analysis, inappropriate empiric antimicrobial therapy administration was significantly less frequent in cases than in comparators (5.5% vs. 38.8%). The 21-day mortality rate was twofold lower in cases than in comparators (22.2% vs. 44.4%), although the difference was not significant. No other analysed variables were significantly different between the two groups.

**Conclusions:**

This study illustrated that T2Dx might be associated with an increase in the appropriateness of empiric antimicrobial therapy in patients with BSI. Further studies are needed to evaluate whether the T2Dx assay can improve patient outcomes.

## Background

Delayed administration of active anti-infective therapy is associated with increased rates of mortality and higher medical costs in patients with severe infections, especially bloodstream infection (BSI) [1, 2]. Despite the recent updates of definitions and clinical criteria [3], early recognition of infection and timely management of patients with sepsis remain challenging, especially in multidrug-resistant microorganism-associated infections and in critical care settings [4].

Antimicrobial-resistant ESKAPE (i.e., *Enterococcus faecium*, *Staphylococcus aureus*, *Klebsiella pneumoniae*, *Acinetobacter baumannii*, *Pseudomonas aeruginosa* and *Enterobacter* species) microorganisms represent a frequent cause of nosocomial BSI [5, 6], and they can acquire antimicrobial resistance genes, thereby reducing treatment options and increasing death rates because of treatment failure [7]. In addition to bacteria, *Candida* species represent an important cause of BSI [8–10], which is often misdiagnosed because of the low sensitivity of conventional methods [11], and they are associated with high lethality rates [8].

Although blood culture (BC) is considered the gold standard for BSI diagnosis and it serves as an indispensable assessment method [12], it is often limited by slow turnaround times or the failure to identify causative pathogens [1, 13]. Moreover, the results of BC may be negative even in cases of severe sepsis, and they can be affected by factors such as concurrent antimicrobial use [14]. Because timely and appropriate antimicrobial treatment has a key role in reducing the risk of poor outcomes [4, 9, 15, 16], the development of minimally invasive, highly sensitive and specific diagnostic tests with a short turnaround time and reasonable cost could significantly improve outcomes in patients with bacterial or fungal BSI [1].

The T2Dx system (T2 Biosystems, Lexington, MA, USA), an automated instrument platform using non-culture T2 magnetic resonance technology to detect nucleic acids and microbial cells directly in whole-blood samples [17], has been approved by the US Food and Drug Administration for the rapid detection of BSI caused by a modified panel of ESKAPE bacterial species (including *Escherichia coli* instead of *Enterobacter* spp., i.e. T2Bacteria Panel) [18] or *Candida* spp. (T2Candida Panel) [19]. T2Dx facilitates the significantly more rapid identification of targeted microbial species causing BSI [18, 19]; however, the clinical impact of T2Dx on the appropriateness of empirical antimicrobial therapy and outcome is unclear [20–22].

In the present single-centre study, our real-world experience of the use of T2Dx in addition to standard BC is described. Moreover, the possible impact of the T2Dx assay on the appropriateness of empiric antimicrobial therapy and clinical outcome was investigated.

## Materials and methods

### Study design, setting and population

Between January 1, 2018 and December 31, 2019, T2Dx (T2Bacteria and T2Candida; T2 Biosystems) was available at the ‘*Mater Domini*’ teaching hospital of Catanzaro, Italy. We performed a retrospective, observational, matched case–control study including all consecutive patients hospitalised during this period with suspected BSI who underwent testing using T2Dx (T2Bacteria and/or T2Candida) in addition to standard BC (T2 group).

T2 group patients were divided into subgroups according to the presence and absence of a T2 result (T2-positive group and T2-negative group, respectively). The criteria for using the T2 method were not defined a priori (as part of specific study protocols), but they were based on the clinical judgement of the patients’ attending physicians or an infectious disease consultant. The results of T2Dx were compared to those of paired standard BC. For each patient enrolled in the T2-positive group, we randomly selected two matched patients (comparators) diagnosed with microbiologically documented BSI by standard BC alone during hospitalisation. Subjects were matched by ward area (i.e. intensive care, medical or surgical) and date of BSI (± 2 months); in addition, only cases of BSI caused by microbial species (at least one in cases of polymicrobial infections) included in the T2Dx panels were selected. Demographic, clinical (including antimicrobial therapies and outcome measured as 21-day mortality) and microbiological records were included in a pre-defined case report form for each patient. T2-positive patients were compared to comparators, and the possible clinical impact of T2Dx on the appropriateness of empirical antimicrobial therapy was evaluated.

The following terms were defined prior to data analysis:

- T2Dx results were considered concordant with BC findings if the same microbial species were identified by both methods and no other bacterial or fungal species were identified by either method; otherwise, the T2Dx and BCs results were considered discordant.
- Empirical antimicrobial therapy was considered to be appropriate if it was started immediately after T2Dx and BC sampling and included at least one antimicrobial drug to which the pathogen(s) responsible for BSI subsequently displayed in vitro susceptibility according to standard methods. In patients with a positive T2Dx result in the absence of supporting culture data, the empirical antimicrobial therapy was considered appropriate based on the in vitro susceptibility phenotype of the same microbial species isolated within 7 days from a bacteriological culture from an extra-blood site (e.g. abdomen, respiratory tract) that was considered the primary source of BSI or if the prescribed antimicrobial therapy displayed clinical efficacy with prompt resolution of the signs and symptoms of infection. For this purpose, each medical record was reviewed independently by two investigators (VS and EMT).

### Microbiological procedures

For each patient, a whole-blood sample for the T2Bacteria Panel (*E. faecium*, *S. aureus*, *K. pneumoniae*, *A. baumannii*, *P. aeruginosa* and *E. coli*) and/or T2Candida Panel (*C. albicans/C. tropicalis*, *C. glabrata/C. krusei and C. parapsilosis*) assays was collected into 4-mL K2EDTA Vacutainer blood collection tubes. BCs samples were collected simultaneously using the same peripheral vein puncture sites. T2 specimens were processed immediately by a fully automated T2Dx instrument based on T2 magnetic resonance detection [17]. None of positive results obtained by T2Dx instrument was confirmed by a second sample, collected immediately after obtaining the result of the first one.

Culturing was performed for 5–7 days in accordance with routine laboratory practice using the BacT/ALERT VIRTUO system (bioMérieux, Florence, Italy). Positive BCs were subjected to our BSI diagnostic flowchart that included Gram staining microscopy integrated with molecular assays (i.e. Filmarray/ePlex) and conventional biochemical tests (Vitek2, bioMérieux) as well as proteomic methods (MALDI-TOF- MS, Shimadzu Scientific Instruments, Columbia, MD, USA).

### Statistical analysis

Continuous variables were compared using Student’s *t*-test (normally distributed variables) or the Mann–Whitney U test (non-normally distributed variables). Categorical variables were evaluated using the chi-squared test or two-tailed Fisher’s exact test. Values are expressed as the mean ± standard deviation (continuous variables) or as percentages of the group from which they were derived (categorical variables). P < 0.05 was considered significant. All statistical analyses were performed using the Intercooled Stata programme, version 16, for Windows (Stata Corporation, College Station, TX, USA).

## Results

### Enrolled patients

**Fig. 1:**
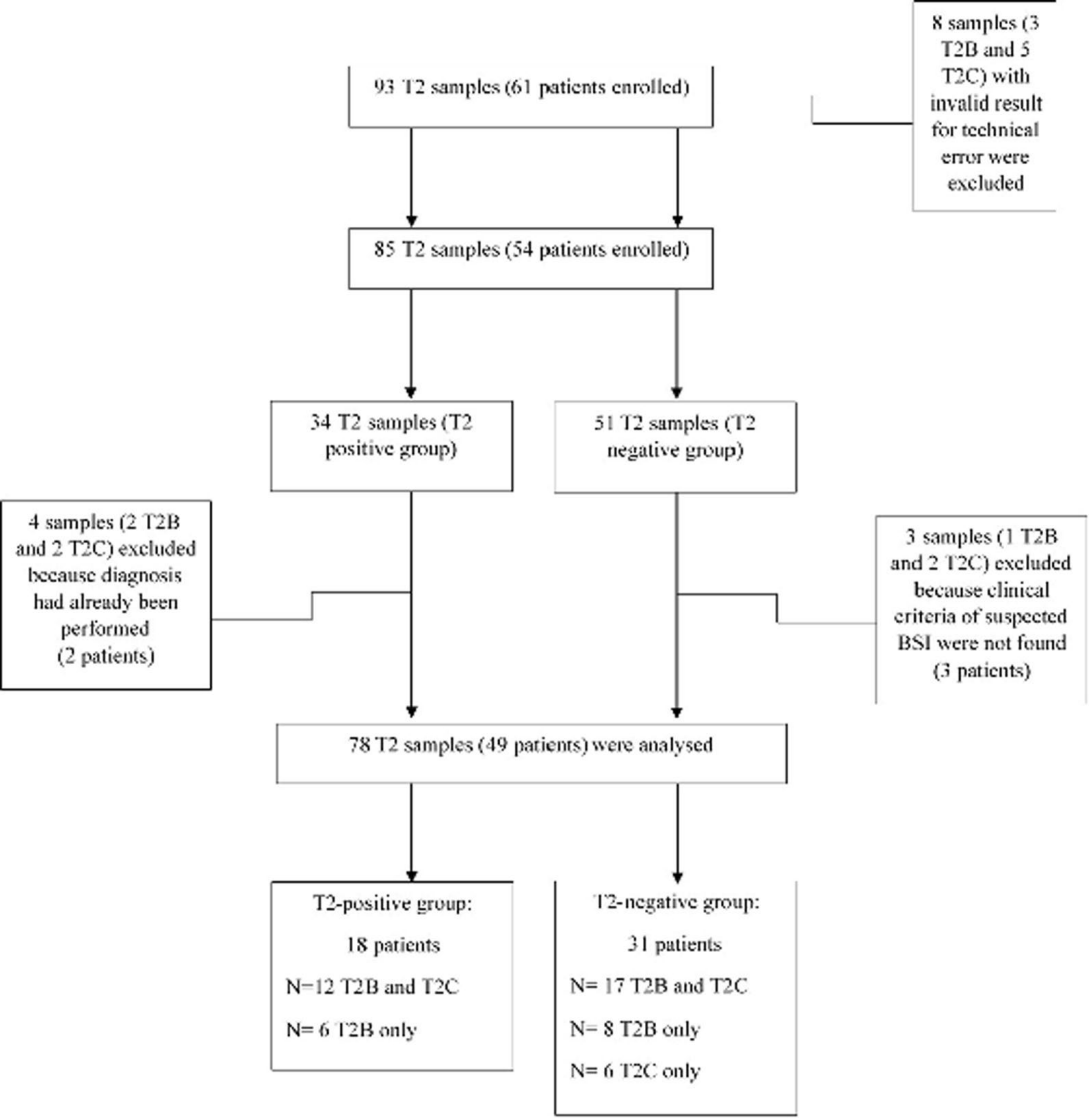
Flowchart of patients and blood samples included in the study. T2B, T2Bacteria; T2C, T2Candida

During the study period, the T2Dx rapid identification test was performed in addition to standard BC for 61 patients. Seven patients were excluded because an invalid result was obtained for the T2Dx assay as a result of technical errors. Therefore, 54 patients were enrolled. The T2Dx assay result was positive for 20 patients and negative for 34 patients. Among the patients with a negative result, three were excluded from the clinical analysis because of the absence of clinical criteria for suspected BSI, whereas two patients with positive results were excluded because the diagnosis had already been confirmed using standard BC. Therefore, the study included 18 T2-positive patients and 31 T2-negative patients. Figure 1 presents the flowchart of the study.

### Microbiological results

In total, 78 samples from 49 patients were analysed (in 29/49 patients, both T2Bacteria and T2Candida panels were performed). Table 1 presents the results from T2Dx and standard BC for both bacteria and fungi.

**Table 1:** Results of T2Dx and standard blood culture for bacteria and fungi

Bacteria			
		T2Dx	Blood culture
Positive T2Dx Negative blood culture	n = 6	*E. faecium* (1)	
		*K. pneumoniae*/*A. baumannii* (1)	
		*A. baumannii* (1)	
		*P. aeruginosa* (2)	
		*E. coli* (1)	
Positive T2Dx Positive blood culture	n = 8	*A. baumannii* (2)	*A. baumannii* (2)
		*E. coli* (1)	*E. coli* (1)
		*K. pneumoniae*/*A. baumannii* (1)	*K. pneumoniae* (1)
		*K. pneumoniae*/*E. coli* (2)	*E. coli* (2)
		*E. faecium* (1)	*Acinetobacter* spp. (1)
		*A. baumannii* (1)	*E. cloacae* complex (1)
Negative T2Dx Positive blood culture	n = 7		*S. epidermidis* (5)
			*S. epidermidis*/*A. baumannii* (1)
			*K. pneumoniae* (1)

Fungi			
		T2Dx	Blood culture
Positive T2Dx Negative blood culture	n = 2	*C. albicans*/*C. tropicalis* (1)	
		*C. parapsilosis* (1)	
Positive T2Dx Positive blood culture	n = 3	*C. albicans*/*C. tropicalis* (2)	*C. albicans* (2)
		*C. albicans*/*C. tropicalis* + *C. parapsilosis* (1)	*C. albicans* + *C. parapsilosis* (1)
Negative T2Dx Positive blood culture	n = 1		*C. albicans* (1)

‘n’ represents the number of tests which also corresponded to the same number of patients. At least one bacterial species was identified in eight patients, and at least one *Candida* spp. was identified in one patient. Cases of complete discordance are listed in boxes with a grey background, cases of incomplete concordance are listed in boxes with a vertical line background and cases of complete concordances are listed in boxes with a white background. The number of isolates is reported in parentheses for each species

### T2Bacteria panel

In total, 43 samples were collected from 43 patients and analysed using the T2Bacteria Panel, and the results of T2Dx and BCs were concordant for 25/43 samples (58.1%). Excluding bacterial species not included in the T2Bacteria Panel and identified via BC (e.g. coagulase-negative *Staphylococci*), the rate of concordance between T2Dx and BC was 32/43 (74.4%). Table 1 presents the bacterial species identified in T2Dx and/or concomitant BC. Both T2Dx and BCs produced negative results for bacterial species in 22 samples.

### T2Candida panel

Meanwhile, 35 samples were collected from the same number of patients and analysed using the T2Candida Panel. The results of T2Dx and BC were concordant for 32/35 samples (91.4%). Table 1 presents the *Candida* species identified using T2Dx and/or concomitant BC. Both standard BC and T2Dx generated negative results for fungal isolates in 32 samples.

### Patients’ clinical results

#### Clinical and demographic characteristics of patients who underwent the T2Dx assay

The demographic and clinical characteristics of 49 patients analysed using the T2Dx identification test in addition to standard BC are presented in Table 2.

**Table 2:** Clinical characteristics and analysed variables of patients for whom the T2Dx assay was performed in addition to standard blood culture

Variables^a^	Total of patients (n = 49)	T2-positive group (n = 18)	T2-negative group (n = 31)
*Demographic information*			
Age (years), mean (SD)	62.3 (16.6)	59.7 (21.1)	63.8 (13.5)
Men	36 (73.4)	13 (72.2)	23 (74.2)
*Patient’s co-morbidities*			
Chronic kidney disease	11 (22.4)	3 (16.6)	8 (25.8)
Hypertension	29 (59.1)	9 (50.0)	20 (64.5)
Diabetes	12 (24.4)	3 (16.6)	9 (29.0)
Ischaemic heart disease	15 (30.6)	4 (22.2)	11 (35.4)
Neurological disease	8 (16.3)	4 (22.2)	4 (12.9)
COPD	4 (8.1)	1 (5.5)	3 (9.6)
Obesity	7 (14.2)	1 (5.5)	6 (19.3)
Cancer (all)	12 (24.4)	6 (33.3)	6 (19.3)
Solid cancer	8 (16.3)	4 (22.2)	4 (12.9)
Haematological cancer	4 (8.1)	2 (11.1)	2 (6.4)
*Hospitalisation area*			
Intensive care unit	31 (63.2)	11 (61.1)	20 (64.5)
Medical unit	14 (28.5)	5 (27.8)	9 (29.1)
Surgical unit	4 (8.1)	2 (11.1)	2 (6.4)
*Primary site of BSI*			
Respiratory	19 (38.7)	8 (44.4)	11 (35.4)
Urinary	5 (10.2)	3 (16.6)	2 (6.4)
Abdominal	9 (18.3)	4 (22.2)	5 (16.1)
Heart	2 (4.1)	0	2 (6.4)
Skin and soft tissue	3 (6.1)	2 (11.1)	1 (3.2)
Unknown	11 (22.4)	1 (5.5)	10 (32.2)
*Hospitalisation data*			
Length of hospitalisation (days), mean (SD)	20.0 (18.8)	16.8 (11.1)	21.8 (22.1)
Time from admission to infection (days), mean (SD)	14.4 (16.5)	16.0 (15.6)	13.5 (17.2)
Time from infection to discharge/death (days), mean (SD)	34.4 (28.3)	32.7 (14.9)	35.3 (33.9)
*Miscellaneous*			
PCT (ng/mL), mean (SD)	19.1 (46.0)	18.6 (42.8)	19.3 (48.4)
Mechanical ventilation	29 (59.1)	10 (55.5)	19 (61.2)
Inotrope drugs	33 (67.3)	9 (50.0)	24 (77.4)
ECMO	9 (18.3)	4 (22.2)	5 (16.1)
CVVH	10 (20.4)	2 (11.1)	8 (25.8)
21-day mortality	15 (30.6)	4 (22.2)	11 (35.4)

*COPD* chronic obstructive pulmonary disease, *PCT* procalcitonin, *ECMO* extracorporeal membrane oxygenation, *CVVH* continuous veno-venous haemofiltration

^a^All values are presented as n (%) unless otherwise specified

These patients, who included 36 men (73.4%), had a mean age of 62.3 ± 16.6 years. The most common co-morbidities were hypertension (29/49; 59.1%) and ischaemic heart disease (15/49; 30.6%).

The most frequent primary site of infection was the respiratory tract (19/49; 38.7%), and the primary site of infection was more frequently unknown in the T2-negative group (10/31; 32.2%) than in the T2-positive group (1/18; 5.5%).

### *T2-positive group (cases) *vs.* matched patients’ group (comparators)*

The demographic and clinical characteristics of the T2-positive and matched patients (comparators) groups are presented in Table 3.

**Table 3:** Clinical characteristics and analysed variables between cases (patients with positive T2Dx results) and matched patients (comparators)

Variables	T2-positive group (n = 18)	Standard blood culture (n = 36)	p value
Demographic information			
Age (years), mean (SD)	59.7 (21.1)	60.2 (16.0)	0.923
Male gender	13 (72.2)	24 (66.6)	0.678
Patient’s co-morbidities			
Chronic kidney disease	3 (16.6)	7 (19.4)	0.804
Hypertension	9 (50.0)	12 (33.3)	0.236
Diabetes	3 (16.6)	6 (16.6)	1.000
Ischaemic heart disease	4 (22.2)	8 (22.2)	1.000
Neurological disease	4 (22.2)	4 (11.1)	0.278
COPD	1 (5.5)	7 (19.4)	0.175
Obesity	1 (5.5)	6 (16.6)	0.251
Cancer (all)	6 (33.3)	12 (33.3)	1.000
Solid cancer	4 (22.2)	8 (22.2)	1.000
Haematological cancer	2 (11.1)	5 (13.8)	0.774
Hospitalisation area			
Intensive care unit	11 (61.1)	20 (55.5)	0.697
Medical unit	5 (27.7)	11 (30.5)	0.833
Surgical unit	2 (11.1)	5 (13.8)	0.774
Microorganism			
*Escherichia coli*	4 (22.2)	10 (27.7)	0.660
*Klebsiella pneumoniae*	4 (22.2)	9 (25.0)	0.821
*Pseudomonas aeruginosa*	2 (11.1)	4 (11.1)	1.000
*Acinetobacter baumannii*	6 (33.3)	11 (30.5)	0.835
*Enterococcus faecium*	2 (11.1)	4 (11.1)	1.000
*Candida spp.*	5 (27.7)	5 (13.8)	0.215
Mixed species	5 (27.7)	6 (16.6)	0.339
Primary site of BSI			
Respiratory	8 (44.4)	16 (44.4)	1.000
Urinary	3 (16.6)	4 (11.1)	0.566
Abdominal	4 (22.2)	8 (22.2)	1.000
Heart	0	1 (2.7)	0.475
Skin and soft tissue	2 (11.1)	3 (8.3)	0.739
Unknown	1 (5.5)	5 (13.8)	0.358
Hospitalisation data			
Length of hospitalisation (days), mean (SD)	32.7 (14.9)	25.1 (17.5)	0.115
Time from admission to infection (days), mean (SD)	16.1 (15.7)	12.3 (15.5)	0.411
Time from infection to discharge/death (days), mean (SD)	16.8 (11.1)	12.8 (11.1)	0.219
Miscellaneous			
PCT at baseline, mean (SD)	18.6 (42.8)	20.6 (37.2)	0.858
Mechanical ventilation	10 (55.5)	17 (47.2)	0.563
Inotrope drugs	9 (50)	17 (47.2)	0.847
ECMO	4 (22.2)	4 (11.1)	0.278
CVVH	2 (11.1)	7 (19.4)	0.438
Inappropriate empiric antibiotic therapy	1 (5.5)	14 (38.8)	**0.009**
21-day mortality	4 (22.2)	16 (44.4)	0.110

*COPD* chronic obstructive pulmonary disease, *PCT* procalcitonin, *ECMO* extracorporeal membrane oxygenation, *CVVH* continuous veno-venous hemofiltration

All values are presented as n (%) unless otherwise specified

There were no significant differences in any variables except that inappropriate empiric antibiotic therapy was less frequently provided to cases than to comparators (1/18; 5.5% vs. 14/36; 38.8%; P = 0.009). Among the patients in the T2-positive group, in 6/17 patients (35.3%) T2Dx was useful for ensuring the prescription of appropriate empiric antimicrobial therapy, which was changed as soon as the test results were available, on average after 4.5 h. For the remaining 11 patients, empiric antimicrobial therapy had already been appropriately prescribed by infectious disease specialist and had been confirmed until BC results were available. The 21-day mortality rate was twofold lower in cases than in comparators (4/18, 22.2% vs. 16/36, 44.4%), although the difference did not reach significance (P = 0.11). The 21-day mortality rate was not significantly different between patients who had received an inappropriate vs. appropriate empirical antimicrobial therapy (33.3%, 5/15 vs. 38.5%, 15/39; P = 0.72).

## Discussion

This study described our real-world experience of the use of T2Dx in addition to BC in a third-level university hospital, focusing especially on the clinical impact of T2Dx on the appropriateness of empirical antimicrobial therapy and patient outcome.

In this study, an apparently low rate of concordance between the T2Bacteria Panel and BC compared to previous studies [22] was observed. Differing from previous studies, our study was not focused on the performance of the T2Dx tests; for this reason, the crude concordance rate between T2Dx and standard BC was reported, and the performance parameters of the T2Dx test were not calculated. Of note, in previous studies of the T2Bacteria test, a ‘true-infection criterion’ was used in which discordant T2Bacteria results were considered concordant results when the same microorganism detected only by T2Bacteria grew from a culture obtained within 7 days from a clinical sample other than blood and it was considered the primary source of BSI [18, 23]. No recommendations about the exact interpretation of T2Dx results in clinical practice are available to date.

The reported sensitivity rates of the T2Candida Panel range 89–100%, which are higher than that of standard BC for candidemia (50%) [20, 24, 25]. In line with previous studies, the rate of concordance between T2Dx and standard BC was high in our experience. Only in one case, candidemia was diagnosed exclusively by BC, being T2Candida panel negative for *Candida* spp.. This result is surprising, in view of the fact that T2Candida panel has been reported to have a high negative predictive value such that a negative result could support early discontinuation of empiric antifungal therapy in ICU patients with suspected candidemia. Therefore, T2Dx should be performed in addition to standard BC, and empirical therapy can be stopped only after a definitive result is obtained for BC if invasive candidemia is suspected [21].

Because a delay in the initiation of appropriate antimicrobial therapy represents a well-recognised risk factor for mortality in patients with bacterial or fungal BSI [9, 15, 16, 26–28], the primary aim of the present study was to investigate the impact of T2Dx on the prescription of appropriate empirical antimicrobial therapy and outcome. Appropriate empirical antimicrobial therapy was prescribed with a significantly higher frequency among patients with a positive T2Dx result than among matched comparator patients, for whom T2Dx was not performed. Interestingly, among patients who underwent the T2Dx assay, the rate of appropriate empirical antimicrobial therapy was increased. Moreover, T2Dx allowed us to identify a breakthrough severe infection by *K. pneumoniae* and *A. baumannii* that resulted in clinical failure in a patient whose concomitant BCs were negative, and a new in vitro active antibiotic (cefiderocol) obtained through the manufacturer’s compassionate use was administered with clinical success [29]. Therefore, it can be speculated that T2Dx could improve the appropriateness of empirical antimicrobial therapy. Conversely, it must be noted that T2Dx was performed outside a clinical trial design in our population. In addition, because T2Dx was mostly performed in more critical patients in our experience, extended-spectrum antibiotic therapy was more frequently prescribed.

Of note, although the 21-day mortality rate was not statistically different between the T2-positive and comparator groups, a twofold difference was recorded between the groups. Considering the well-recognized impact of inappropriate empirical antimicrobial (either antibiotic or antifungal) therapy on mortality in patients with BSI, it is possible that the higher rate of appropriate empiric antimicrobial therapy in the T2-positive group help to reduce 21-day mortality but that a statistical difference was not observed because of the small size of our study population. On the other hand, in this study, the 21-day mortality rate did not result significantly different between patients who were treated with appropriate vs. inappropriate empirical antimicrobial therapy. This could be due to several factors: first, the small number of patients included; second, the severity of BSI in cases leading to the indication of making a rapid diagnosis using T2Dx may have been greater than in the comparators (although in such a case this ‘confounding by indication’ bias could have diluted the impact of the study test on the clinical outcome); third, the cohort included patients with microbiologically documented BSI caused both by bacteria or *Candida* spp., the prognosis of which is indeed different; four, the role of source control was not investigated.

This study was affected by some limitations. In particular, the small population size and the retrospective nature of the study reduced the generalisability of the results. Specifically, cases and comparator patients were not matched for disease severity.

In conclusion, T2Dx appeared to be a highly sensitive and specific diagnostic test with a short turnaround time and the potential to improve outcomes in patients with BSI. To our knowledge, this is the first study to demonstrate that T2Dx facilitated a statistically significant increase in the rate of appropriate empiric antibiotic therapy and potentially reduced mortality among patients with BSI. We believe that prospective randomised studies are needed to validate these findings. Meanwhile, because no clear recommendations on its use in real clinical practice have been posed and its clinical utility is uncertain [30], T2Dx should be used as complement to standard BC, especially in patients with more severe prognoses.

## Data Availability

Data are available upon request from the corresponding author.
